# Study of the variation of the *Malassezia* load in the interdigital fold of dogs with pododermatitis

**DOI:** 10.1007/s11259-022-09951-2

**Published:** 2022-06-15

**Authors:** Leyna Díaz, Gemma Castellá, M. Rosa Bragulat, Andreu Paytuví-Gallart, Walter Sanseverino, F. Javier Cabañes

**Affiliations:** 1 Veterinary Mycology Group, Department of Animal Health and Anatomy, Universitat Autònoma de Barcelona, Bellaterra, Catalonia Spain; 2 Sequentia Biotech SL, Barcelona, Catalonia Spain

**Keywords:** Interdigital fold, *Malassezia*, *M. pachydermatis*, Mycobiome, Pododermatitis

## Abstract

The yeast *Malassezia pachydermatis* is a common inhabitant of the skin and mucosae of dogs. However, under certain circumstances this yeast can overgrow and act as an opportunistic pathogen causing otitis and dermatitis in dogs. Canine pododermatitis is a common disorder in dogs in which *M. pachydermatis* acts as an opportunistic pathogen. In the present study, the presence of *Malassezia* yeasts was assessed and quantified in samples collected from the interdigital space of dogs with pododermatitis before and after treatment, and from healthy dogs. The samples were subjected to two different cytological examinations, culture on Sabouraud glucose agar and modified Dixon’s agar and a quantitative PCR targeting the internal transcribed spacer (ITS) genomic region. A selection of samples was analyzed by next generation sequencing (NGS) using the D1D2 domain of the large subunit of the ribosomal DNA as target. The pododermatitis samples before treatment showed higher cell counts, colony-forming units and ITS copies than the rest of samples. The NGS analysis revealed that *Ascomycota* was the main phylum in the healthy and post-treatment samples. However, *Basidiomycota* and *M. pachydermatis* was more abundant in the pododermatitis samples before treatment. These results support *M. pachydermatis* as an opportunistic agent in canine pododermatitis by a variety of methods, and demonstrate the correlation between cytologic and molecular methods for quantification.

**Supplementary Information:**

The online version contains supplementary material available at 10.1007/s11259-022-09951-2.

## Introduction

The yeast *Malassezia pachydermatis* is part of the normal microbiota of the skin and mucosae of healthy dogs (Guillot and Bond 1999; Cabañes 2021). Despite being part of the normal microbiota, under certain circumstances, the population of *M. pachydermatis* can overgrow and this yeast may act as an opportunistic pathogen. Thus *M. pachydermatis* is considered one of the most frequent aetiological agents responsible for otitis and dermatitis in dogs (Bond et al. 2020).

Canine pododermatitis is a common disorder in the general veterinary practice*. Malassezia pachydermatis* commonly acts as an opportunistic pathogen that produces pododermatitis in dogs, especially in atopic animals or animals diagnosed with endocrine disease. It may affect the interdigital spaces, nail folds, and nails, and usually more than one paw is affected (Duclos 2013; Bajwa 2016). In dogs with *Malassezia* pododermatitis, pruritus is a major and constant sign often associated with erythema and/or greasy exudate. In chronic cases hyperpigmentation, alopecia and lichenification may be present (Miller et al. 2000).

Its diagnosis is based on history, physical examination, clinical signs, cytological examination, and response to antifungal therapy (Miller et al. 2000; Bond et al. 2010). Detection of *Malassezia* yeasts by cytology in compatible skin lesions is a key for the diagnosis of *Malassezia* pododermatitis (Duclos 2013). Cultural techniques are primarily used in research rather than routine veterinary practice. However, they have been suggested to have a diagnostic value because they have higher sensitivity than cytology (Bond et al. 1994, 2010).

To overcome limitations of culture-based techniques, several molecular methods have been developed. To detect and quantify *Malassezia* yeast in samples, quantitative PCR (qPCR) is a fast, sensitive, and precise technique (Sugita et al. 2006). The internal transcribed spacer (ITS) region and β-tubulin gene have been used as target to study the population of *Malassezia* in samples from dogs by qPCR (Puig et al. 2019; Meason-Smith et al. 2020).

On the other hand, next generation sequencing (NGS) is a high sensitivity tool to investigate the microbial diversity within samples at a lower cost without the need of a culture (Forde and O’Toole 2013; Forbes et al. 2017). Although only few studies have been conducted using NGS in dogs, Tang et al. (2020) highlighted the adequacy of NGS-based methods as a diagnostic tool to diagnose canine skin and ear infections.

The aim of this work was to study the presence of *M. pachydermatis* from samples of dogs with pododermatitis, healthy dogs and dogs after treatment using different techniques including cytologic examination, culture, qPCR and NGS, and to determine if there is a correlation between common cytological techniques used in veterinary practice and molecular methods. Besides, using NGS the mycobiome was compared among a selection of the three different kind of samples studied.

## Materials and methods

### Animals and sample collection

A total of 13 dogs including five females and seven males were enrolled in this study (Table 1). Eleven dogs were included in the pododermatitis group. All were diagnosed with pruritus in at least one extremity, erythema in the affected interdigital fold, and a positive cytology for the presence of *Malassezia* yeasts. A total of 15 samples were collected from this group. Three dogs initially diagnosed with *Malassezia* pododermatitis were also sampled after treatment with antifungal agents (Table 1). These dogs were included in the post-treatment group and four samples were obtained. Two dogs without erythema, pruritus and skin lesions were assigned to the healthy group, obtaining three samples. A total of 22 samples were obtained following the procedures on Animal and Human Experimentation from UAB and Generalitat de Catalunya approved by Ethics Committee (Approval number, 4600; Approval date, 22Feb2019). Also, the written consent of owners was obtained before sampling the animals.

**Table 1:** Samples included in the study

Sample	Dog	Breed	Sex	Origin	Clinical signs	Treatment
Samples from dogs with pododermatitis						
**1**	Dog 1	Labrador Retriever	FN	Left thoracic limb	Pruritus, erythema, alopecia, and surface debris	_
2	Dog 2	Yorkshire	F	Left thoracic limb	Pruritus, erythema, alopecia, and surface debris	_
**3**	Dog 3	Labrador Retriever	ND	Right pelvic limb	Pruritus, erythema, and greasy exudate	_
**4**	Dog 4	Labrador Retriever	M	Right pelvic limb	Pruritus, erythema, and alopecia	_
5	Dog 5	Bernese Mountain Dog	M	Left thoracic limb	Pruritus, erythema, alopecia, and a small nodule	_
6	Dog 5	Bernese Mountain Dog	M	Right thoracic limb	Pruritus, erythema, and alopecia	_
7	Dog 6	Mixed breed	M	Right thoracic limb	Pruritus, erythema, and greasy exudate	_
8	Dog 7	American Staffordshire	FN	Right pelvic limb	Pruritus, erythema, and alopecia	_
9	Dog 8	Mixed breed	MN	Left thoracic limb	Pruritus and erythema	_
10	Dog 8	Mixed breed	MN	Right thoracic limb	Pruritus and erythema	_
11	Dog 9	West highland white terrier	M	Left thoracic limb	Pruritus, erythema, greasy exudate, and surface debris	_
12	Dog 9	West highland white terrier	M	Right thoracic limb	Pruritus, erythema, greasy exudate, and surface debris	_
13	Dog 10	Labrador Retriever	M	Left thoracic limb	Pruritus, erythema, alopecia, and surface debris	_
17	Dog 13	Labrador Retriever	FN	Right thoracic limb	Pruritus and erythema	_
18	Dog 13	Labrador Retriever	FN	Left thoracic limb	Pruritus and erythema	_
Samples from healthy dogs						
14	Dog 11	Labrador Retriever	MN	Right pelvic limb	None	_
15	Dog 11	Labrador Retriever	MN	Left pelvic limb	None	_
**16**	Dog 12	Labrador Retriever	FN	Right pelvic limb	None	_
Samples after antifungal treatment						
**19**	Dog 3	Labrador retriever	ND	Right pelvic limb	None	Topical clotrimazole twice daily, 4 weeks
20	Dog 7	American Staffordshire	FN	Right pelvic limb	None	Topical miconazole, 3 weeks
21	Dog 8	Mixed breed	MN	Left thoracic limb	None	Oral itraconazole once daily,3 weeks
22	Dog 8	Mixed breed	MN	Right thoracic limb	None	Oral itraconazole once daily, 3 weeks

^a^In bold, samples analysed by NGS. F = female, M = male, FN = neutered female, MN = neutered male, ND = no data available

Two samples were taken from each interdigital fold by simultaneously rotating fully two sterile swabs (Deltalab, Spain) for 30 s. Also, a cell tape (Scotch, Spain) for cytological examination was obtained from each interdigital fold. Tape strip was applied to the skin and then stained with a Diff-Quick staining (Microptic, Spain) of which the first fixative step with methanol was omitted. One swab was used for *Malassezia* culture and cytological examination. This swab was dipped for 10 s into an Eppendorf (Deltalab, Spain) containing 500 µL of a solution of distilled water and 0.04% Tween 80 (ICN Biomedical, USA) to reduce clump formation. This suspension was used to perform cytological examinations and culture. The other swab was maintained at -20 ºC and used for qPCR and NGS.

### Culture and cytological examination

Aliquots of 0.1 mL of the suspension were inoculated on Sabouraud glucose agar (SGA; Oxoid, Spain) and modified Dixon agar (mDA) (Guého et al. 1996) plates using the surface-spread method. Both media were supplemented with 0.05% of chloramphenicol (Sigma, Spain) and 0.05% of cycloheximide (Sigma, Spain) (Guého-Kellerman et al. 2010). The plates were incubated at 35 ºC to a maximum of 7 days and colony forming units (CFU) were counted.

A first cytological examination was performed using the tape strip. A second cytological examination was performed from the suspension. An aliquot of 10 µL of the suspension was heat-fixed on a slide and stained with the Diff-Quick stain. The presence of *M. pachydermatis* cells was quantified by counting cells in 10 different random fields at 100 × with immersion oil.

### DNA extraction

DNA was extracted from swabs using the DNeasy PowerSoil Kit (Qiagen, Germany) according to manufacturer’s instructions with some modifications previously described (Díaz et al. 2021). To control cross-contamination, a sterile swab was processed under the same conditions. The DNA was stored at -20 ºC until used as template for qPCR and NGS.

### *Malassezia pachydermatis* quantification by qPCR

All samples were selected for *M. pachydermatis* quantification. All qPCRs were performed using an Applied Biosystems 7500 real-time system, and SYBR Green chemistry (PowerUp SYBR Green master mix, Applied Biosystems, USA) with the absolute quantification method.

The multicopy ITS region was used as target gene according to Meason-Smith et al. (2020) with some modifications. Primers ITS-ANA-F and PachyR (Vuran et al. 2014) were used at a final concentration of 900 nM each. The runs were performed following these thermal conditions: 1 cycle of 50 ºC for 2 min, 1 cycle of 98 ºC for 3 min, followed by 40 cycles of 95 ºC for 30 s, and 60 ºC for 1 min. Finally, a melting curve analysis with a gradual increase of temperature from 60 ºC to 95 ºC was performed. To achieve absolute quantification of the samples, the standard curve method was used. A standard curve of genomic DNA extracted from the neotype strain of *M. pachydermatis* CBS1879 was constructed, including 7 ten-fold dilutions, from 18 ng to 0.018 pg of DNA. The quantity and purity of the gDNA were determined by spectrometry (NanoDrop 2000; Thermo Scientific, Spain). The number of copies of the template was calculated considering the amount of a template present and the length of the template. All samples were run in duplicate, including the standards and negative control. The negative control contained all the elements of the reaction mixture and water instead of DNA template spiked.

### NGS and data analysis

Due to the high technical cost of the analysis, five samples were selected for the metagenomics NGS analysis of the fungal 26S rRNA gene. These samples were selected based on dog’s breed and the results of the cytological examination and culture. The samples belonged to four different Labrador Retriever dogs. One sample from the healthy group (sample 16) with negative cytology and no growth in the culture media used was included. Three different samples from pododermatitis group (samples 1, 3 and 4) with a positive cytological examination and growth on both culture media used were selected. Also, a sample from the post-treatment group (sample 19) was included.

Quality control was performed at IGA Technology. DNA concentration was evaluated by using a Qubit 2.0 Fluorometer (Invitrogen, USA). Amplicon-seq libraries of D1/D2 regions of the fungal 26S rRNA gene were obtained from each sample by following 16S Metagenomic Sequencing Library Preparation protocol with minor modifications. Briefly, the forward primer (NL1) and the reverse primer (NL4) (Reynolds and Taylor 1993) containing Illumina overhang sequences necessary for the compatibility with Illumina index and sequencing adapters were used for the first PCR amplification under the following conditions: 95 °C for 3 min; 28 cycles of: 95 °C for 30 s, 55 °C for 30 s, 72 °C for 30 s; 72 °C for 5 min; hold at 4 °C. Upon the clean-up, the second PCR was performed under the following conditions: 95 °C for 3 min; 9 cycles of: 95 °C for 30 s, 55 °C for 30 s, 72 °C for 30 s; 72 °C for 5 min; hold at 4 °C. Relevant flow-cell binding domains and unique indices (NexteraXT Index Kit, Illumina, USA) were integrated to the amplicon target. Libraries were normalized by Qubit 2.0 Fluorometer, pooled and sequenced on MiSeq using paired 300‐bp reads, and MiSeq v3 reagents (Illumina). Sequence reads were analyzed in the cloud bioinformatics platform GAIA (https://metagenomics.sequentiabiotech.com) (Giampaoli et al. 2020). Sequencing quality was assessed using FastQC (bioinformatics.babraham.ac.uk/projects/fastqc/) and BBduk (jgi.doe.gov/data-and-tools/bbtools/), setting a minim length of 35 bp and a minimum Phred-quality score of 25.

The resulting high-quality reads were mapped against a custom-made cured database of 26S fungal sequences from NCBI (Díaz et al. 2021). For the taxonomic classification, the mapping-based approach against the database with the BWA mapper (Li and Durbin 2010) was followed by an in house Lowest Common Ancestor (LCA) algorithm. Following the limits proposed by GAIA software, the minimum identity thresholds applied to classify the reads into different taxonomic levels were species (99%), genus (98%), family (96%), order (94%), class (92%), and phylum (90%). Those taxa with abundance below 0.01% considering its mean across the different samples within an experimental group were filtered out for further analysis. DESeq2 (v1.26) (Love et al. 2014) was used to carry out differential abundance analyses. GAIA also assesses the diversity within (alpha-diversity) and between (beta-diversity) samples. Alpha and beta diversities were calculated using the R package phyloseq (McMurdie and Holmes 2013). A Principal Coordinates Analysis (PCoA) was also carried out using Bray–Curtis dissimilarities; cluster significance was assessed with PERMANOVA and ANOSIM (R package vegan).

### Statistical analysis

Other statistical analyses were conducted by Minitab 17 statistical software (Minitab). The Ryan-Joiner normality test was applied to determine whether data followed a normal distribution. Differences in both cytologic examinations, CFU in SGA and mDA, and qPCR quantification values between samples were tested by Kruskal–Wallis test. Student’s t test was applied in values of CFU in both culture media. A multiple regression analysis was used to study the relationship between the qPCR quantification values and values obtained in the rest of variables. The significance level for all statistical analyses was set at *P* ≤ 0.05.

## Results

### Culture and cytological examination

The results of the two cytological examinations performed (tape strip and heat-fixed slide) and the results obtained in both SGA and mDA culture media are summarized in Table 2.

**Table 2:** Results obtained in samples, including the two cytological examinations performed presented in counted cells per field, the colony forming units (CFU) per plate in both Sabouraud’s glucose agar (SGA) and modified Dixon agar (mDA), and qPCR quantification values

Sample	Dog	Cytological examination (cells/field)		Culture CFU		qPCR
		Tape strip	Heat-fixed slide	SGA	mDA	ITS nº of copies
Samples from dogs with pododermatitis						
**1**	Dog 1	1.3	1.1	79	66	2.6 × 10^7^
2	Dog 2	0.2	0	1	0	1.4 × 10^6^
**3**	Dog 3	12.6	0.4	218	1	1.2 × 10^7^
**4**	Dog 4	1.4	0.5	76	14	7.5 × 10^6^
5	Dog 5	6.2	2.2	2	9	2.1 × 10^8^
6	Dog 5	11.4	0.6	120	311	8.1 × 10^7^
7	Dog 6	8.4	0.2	4	0	1.7 × 10^7^
8	Dog 7	45.9	0.7	468	153	2.7 × 10^8^
9	Dog 8	6.8	0.3	172	91	1.3 × 10^6^
10	Dog 8	1.3	0.3	10	17	1.6 × 10^6^
11	Dog 9	4.7	0.2	38	15	1.6 × 10^8^
12	Dog 9	2.3	0	3	1	1.9 × 10^7^
13	Dog 10	4.7	1.3	59	24	2.8 × 10^8^
17	Dog 13	3.2	0	2	1	2.2 × 10^6^
18	Dog 13	0.1	0	5	1	6.5 × 10^6^
Samples from healthy dogs						
14	Dog 11	0	0	0	0	4.9 × 10^6^
15	Dog 11	0	0	0	0	1.1 × 10^5^
**16**	Dog 12	0	0	0	0	1.2 × 10^6^
Samples after antifungal treatment						
**19**	Dog 3	0.2	0	0	0	1.3 × 10^7^
20	Dog 7	0	0	0	0	3.7 × 10^5^
21	Dog 8	0	0	0	0	6.5 × 10^5^
22	Dog 8	0	0	0	0	1.7 × 10^5^

^a^In bold, samples analysed by NGS

The 15 samples from the pododermatitis group had a positive cytological evaluation for *Malassezia* yeast cells, with a range of 0.1–45.9 cells/field (mean = 7.37) on the tape strip. All samples had growth on SGA plates ranging from 1 to 468 CFU/plate (mean = 83.8) (Tables 2 and 3).

**Table 3:** Mean values, standard deviation (SD), and range of values for plate counts (CFU) in Sabouraud’s glucose agar (SGA) and modified Dixon agar (mDA), cytologic examination (cells/field) and qPCR quantification values between samples from dogs with sings of pododermatitis and samples from healthy and post-treatment dogs

	Dogs with pododermatitis (*n* = 15)		Healthy dogs (*n* = 3)		Dogs after treatment (*n* = 4)		*P* value
	mean value ± SD	range	mean value ± SD	range	mean value ± SD	range	
CFU in SGA	83.8 ± 126.0	1.0–468.0	0 ± 0	0–0	0 ± 0	0–0	0.001
CFU in mDA	46.9 ± 85.0	0–311.0	0 ± 0	0–0	0 ± 0	0–0	0.004
Tape-strip	7.37 ± 11.34	0.1–45.9	0 ± 0	0–0	0.05 ± 0.10	0–0.2	0.001
Heat-fixed slide	0.52 ± 0.61	0–2.2	0 ± 0	0–0	0 ± 0	0–0	0.015
qPCR	7.2 × 10^7^ ± 1.0 × 10^8^	1.3 × 10^6^–2.810^8^	2.0 × 10^6^ ± 2.5 × 10^6^	1.1 × 10^5^–4.9 × 10^6^	3.6 × 10^6^ ± 6.4 × 10^6^	1.7 × 10^5^–1.3 × 10^7^	0.014

The three samples from the healthy group had a negative cytological evaluation for the presence of *Malassezia* yeasts and no growth was observed in any of the culture media used.

In the post-treatment group, no growth of *M. pachydermatis* was obtained. All samples had a negative cytological evaluation except for sample 19 which showed a low cell count in the first cytological examination.

Although in almost all samples the SGA medium gave higher CFU counts than mDA, no significant differences (*P* = 0.382) were obtained. Statistically significant differences in tape-strip, heat-fixed slide, plate counts in SGA and mDA media values were found between samples from dogs with pododermatitis signs and healthy and post-treatment dogs (Table 3).

### Quantification of *M. pachydermatis* by qPCR

The standard curve generated was linear over 7 ten-fold dilutions of *M. pachydermatis* DNA, from 1.2 × 10^12^ to 1.2 × 10^5^ copies. The qPCR amplifications of each standard showed amplification plots corresponding to mean Cq values of 14.36 to 34.33. The standard curve yielded *r*^*2 *^values > 0.99, and slope values of − 3.44. Amplification was obtained in all samples with a Cq value lower than 35. The Cq values of samples are detailed in Supplementary Table 1.

As shown in Table 2, in the pododermatitis group, quantification values of 1.3 × 10^6^ to 2.8 × 10^8^ copies were obtained whereas values from 1.1 × 10^5^ to 4.9 × 10^6^ copies were obtained from the healthy group. Samples from the post-treatment group showed quantifications values of 1.7 × 10^5^ to 1.3 × 10^7^ copies. Statistically significant differences in qPCR values were found between samples from the pododermatitis group and samples from the healthy and after treatment groups (Table 3).

Multiple regression analysis showed that the relationship (*r*^*2*^ = 70.7%) between qPCR quantification and both cytological examinations in the model was statistically significant (*P* < 0.001).

### NGS data analysis

The five samples were correctly sequenced, and the generated fastq files reported an average value of 62,178 LSU reads passing filter. The number of generated sequences reads of each sample is described in Table 4. The raw sequencing data is available at the NCBI database (SRA accession number: PRJNA742914).

**Table 4:** NGS reads after filter and biodiversity data obtained from metagenomics analysis

Sample	Number reads after quality processing	% Reads classified to genus	Shannon species diversity	Number of species identified
16 (healthy)	52,488	52.19	3.32	220
1 (pododermatitis)	73,732	26.15	2.37	247
3 (pododermatitis)	60,227	21.58	1.46	201
4 (pododermatitis)	62,178	26.23	2.35	219
19 (post-treatment)	106,095	45.07	2.48	248

The Shannon diversity index was selected to measure alpha diversity. The sample of the healthy dog was the most diverse with an index of 3.32 (Table 4). Samples from the pododermatitis group showed less diversity, on average, than the healthy and post-treatment samples.

The Bray Curtis index was used to measure beta diversity. Samples from the healthy and post-treatment group with an index of 0.323 were more similar compared to the pododermatitis samples (average of 0.716 between the healthy sample and average of 0.496 between the post-treatment sample)*.* Also, pododermatitis samples were more similar among them with an index ranging from 0.112 to 0.196. Although in the PCoA plots, the samples clustered in two different groups, one including the healthy and post-treatment samples and one including all three pododermatitis samples, there was no significant difference between the two different categories based on PERMANOVA ( *F* = 18.9, *P* = 0.1) and ANOSIM (*R* = 1, *P* = 0.1) tests probably due to the small sample size.

**Fig. 1:**
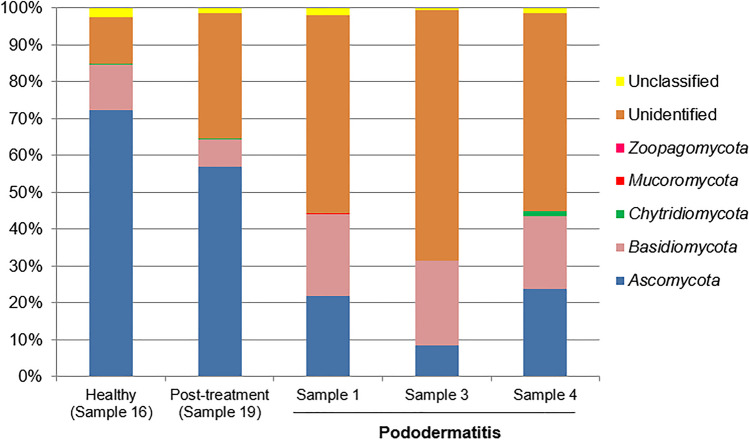
Relative abundance of fungal phyla across different interdigital fold samples

**Fig. 2:**
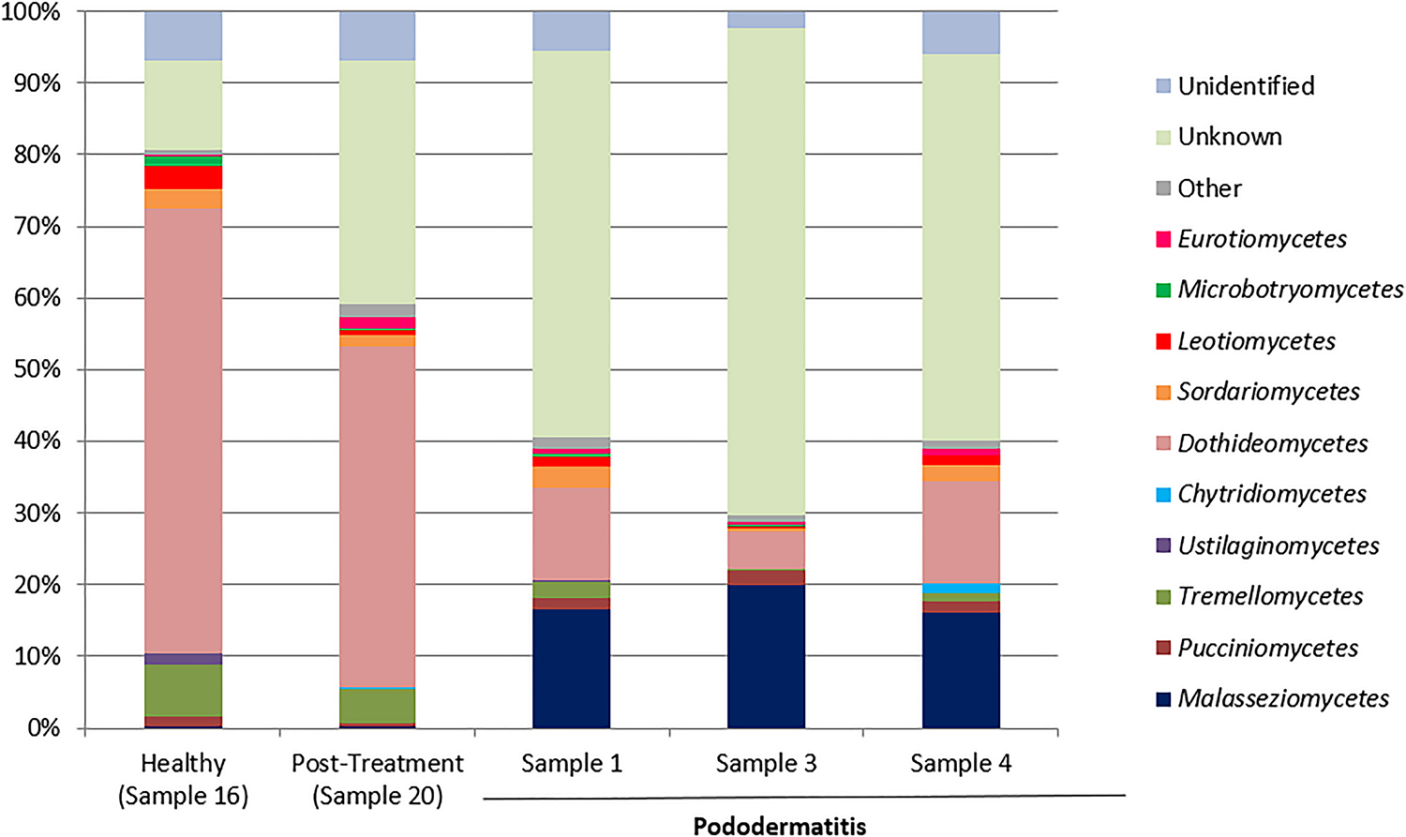
Relative abundance of fungal classes across the different interdigital fold samples

**Fig. 3:**
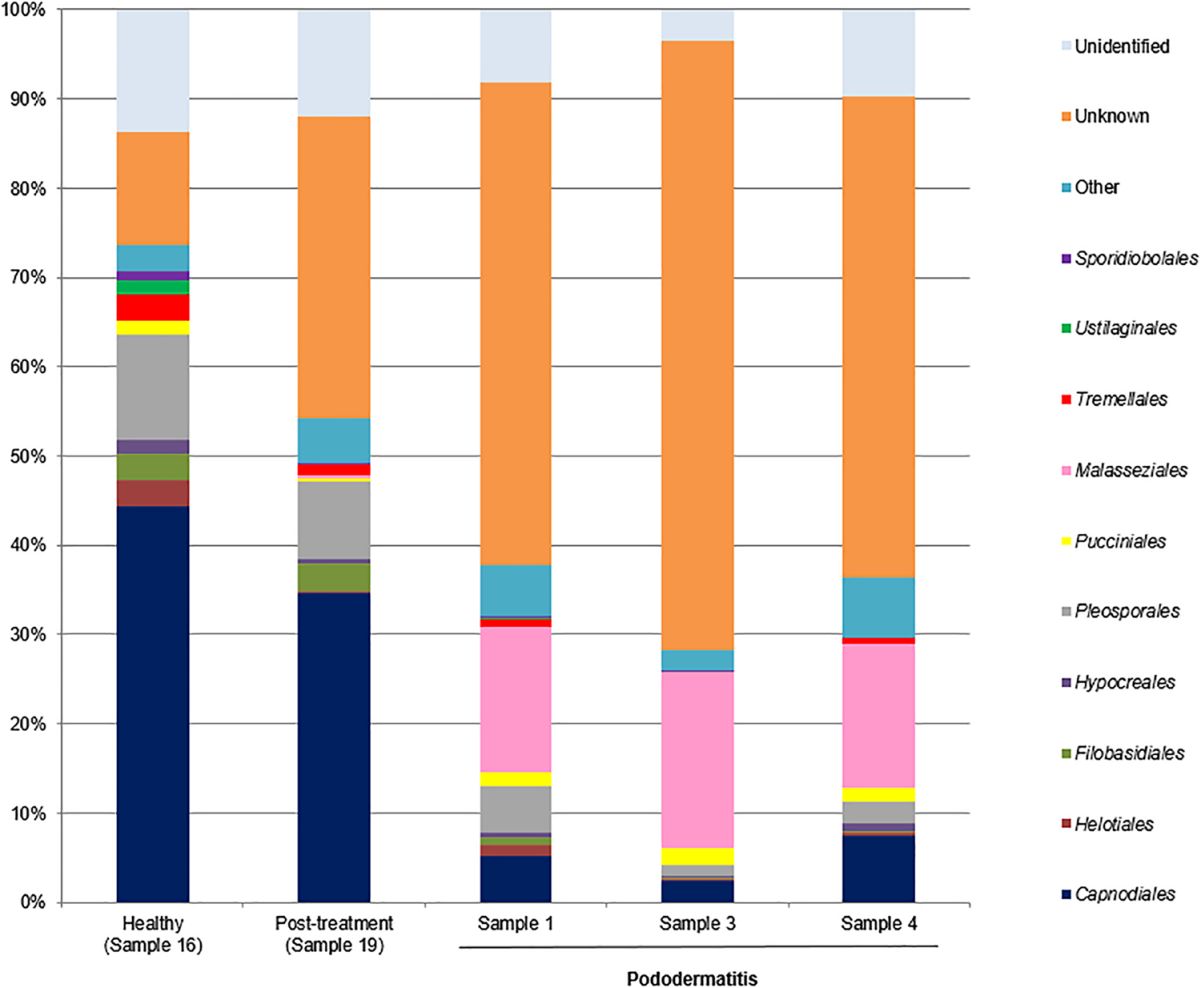
Relative abundance of fungal orders across the different interdigital fold samples

**Fig. 4:**
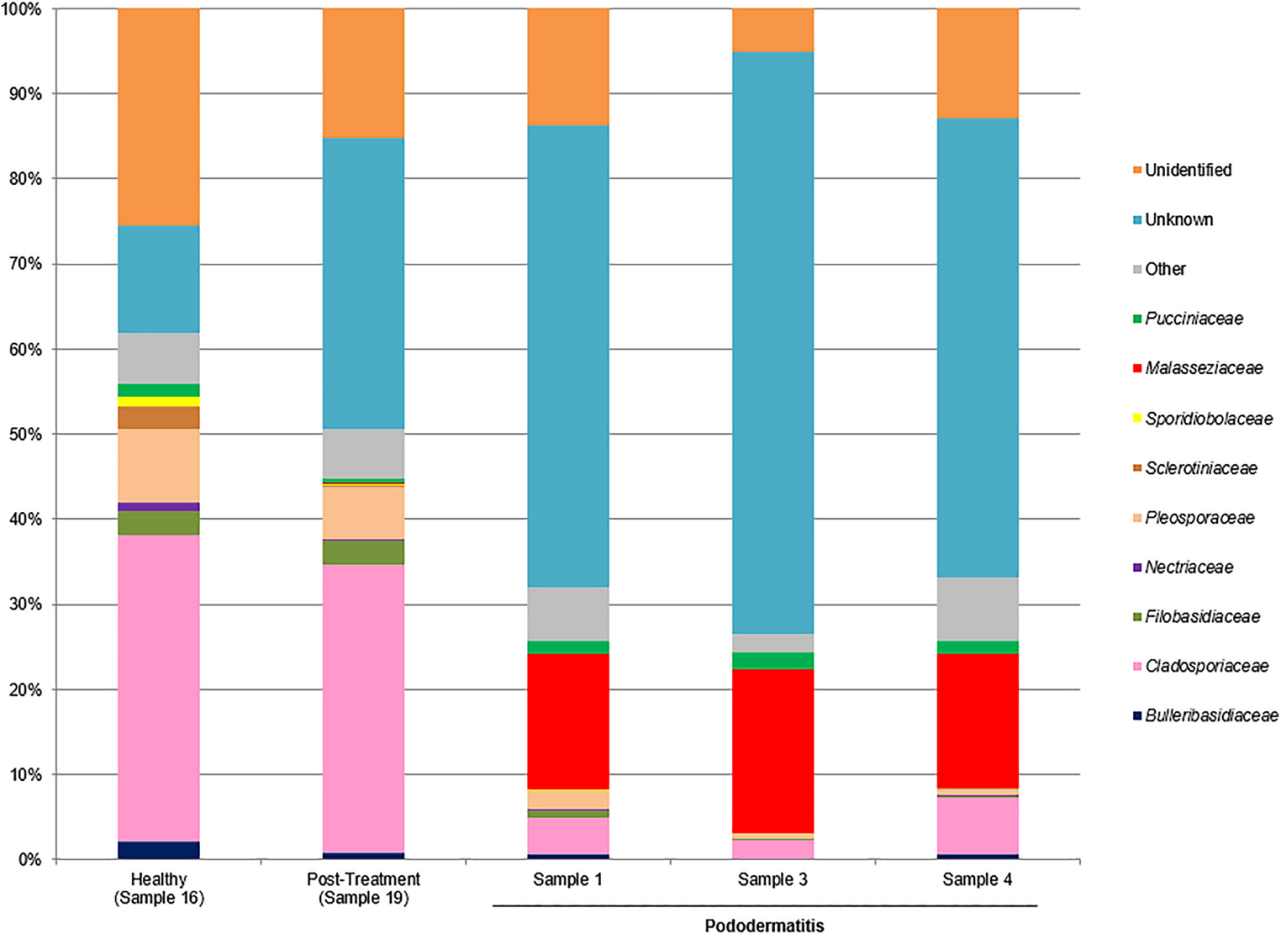
Relative abundance of fungal families across the different interdigital fold samples

**Fig. 5:**
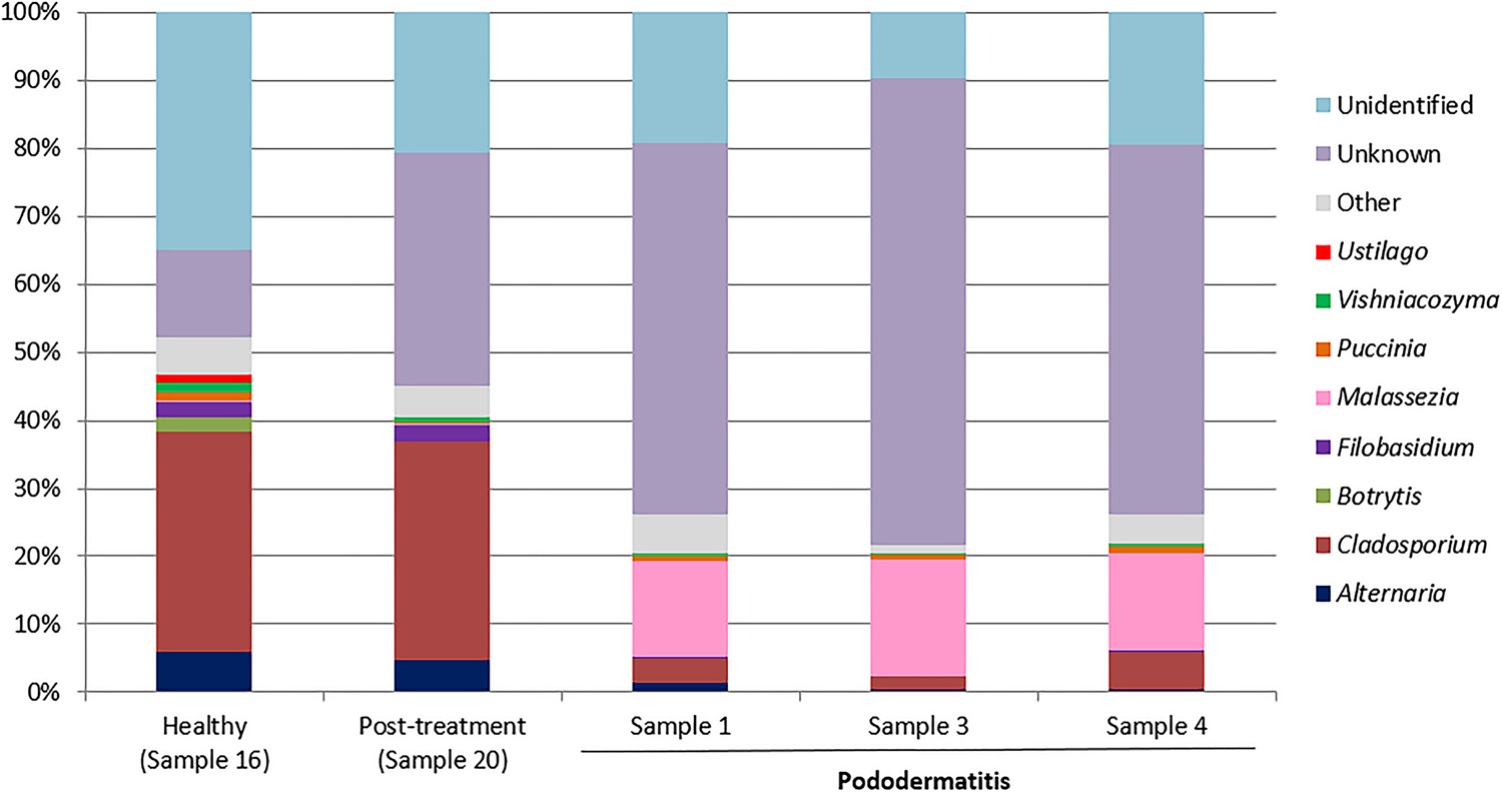
Relative abundance of fungal genera across the different interdigital fold samples

**Fig. 6:**
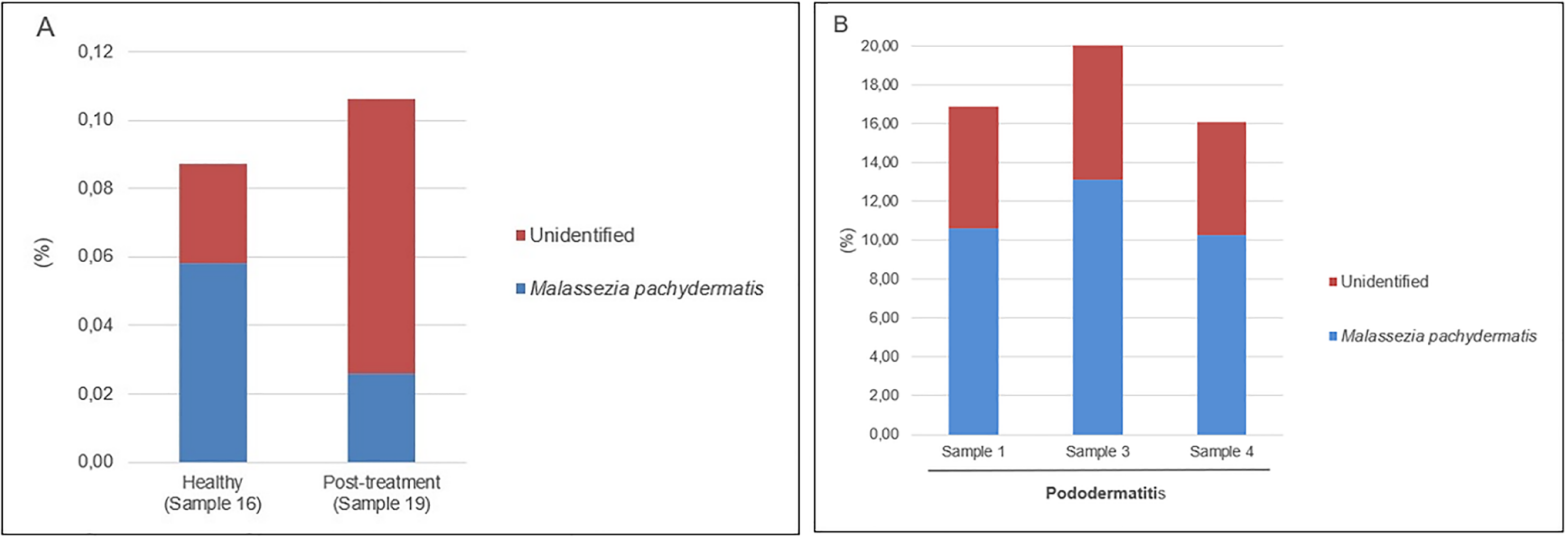
Relative abundance of *M. pachydermatis* and unidentified taxa across the different samples. (**A**) Healthy and post-treatment samples. (**B**) Pododermatitis samples

The taxonomic composition of the samples was investigated at various taxonomic levels. Within the pododermatitis group, *Basidiomycota* with a median of abundance of 22.18% was the main phylum (Fig. 1). At the level of class (Fig. 2), *Malasseziomycetes* showed the highest abundance (16.57%) being *Malasseziales* (16.33%) the main order (Fig. 3). With a median of abundance of 15.94%, *Malasseziaceae* was the main family (Fig. 4). *Malassezia* was the main genus with a median of abundance of 14.26% (Fig. 5) and, *M. pachydermatis* was the predominant species (10.64%). To a lesser extent, other *Malassezia* species were identified in this group of samples like *M. restricta* (0.04%), *M. globosa* (0.02%), and *M. sympodialis* (0.01%). An average of 6.29% of the *Malassezia* sequences could not be identified to the species level (Fig. 6). This percentage includes sequences marked as unknown (6.04%) because a match with enough coverage and identity was not found in our database. Also, the percentage includes ambiguous sequences (0.21%) because they matched with two different sequences from the database but could not discriminate between them. *Malassezia pachydermatis* and *M. sympodialis* showed a higher abundance in the pododermatitis group (*P* < 0.05) while no differences were observed in the abundance of *M. restricta* (*P* = 0.024), and *M. globosa* (*P* = 0.56) between both groups.

Within the healthy and post-treatment samples, *Ascomycota* was the predominant phylum with an abundance of 72.42% and 56.97% respectively (Fig. 1), and *Dothideomycetes* was the main class (Fig. 2). *Capnodiales* with an abundance of 44.54% and 34.64% respectively, was the predominant order (Fig. 3). At the family level (Fig. 4), *Cladosporiaceae* showed the highest abundance in both samples (36.02 and 33.81% respectively) and *Cladosporium* was the main genus with an abundance of 32.40% and 32.15% respectively (Fig. 5). *Malassezia pachydermatis* (Fig. 6) was also detected but with low abundance (0.06% and 0.03% respectively). Other *Malassezia* species were identified in these samples. *Malassezia restricta* (0.02%) was identified in both samples, and *M. globosa* (0.04%) was only present in the post-treatment sample. The percentage of *Malassezia* sequences that could not be identified to the species level in healthy samples decreased to 0.03% whereas in post-treatment samples was 0.08%.

## Discussion

Samples of the pododermatitis group showed the highest number of yeast cells in the two cytological examinations and the highest number of CFU in the plates. Whereas samples from the healthy and after treatment groups showed much less counted cells in both cytological examinations and no growth was observed in the plates. Clinical signs are associated to a hypersensitivity reaction against the yeast. The proliferation of *Malassezia* within the stratum corneum produces numerous antigens and allergens that activate an immune response resulting in epidermal damage and inflammation (Bond et al. 2020). Thus, low yeast counts can produce severe clinical sings and any number of the yeast may be considered significant (Bond et al. 2020). However, in the veterinary practice more than 3–5 cells or more per high power field in the skin or a number higher than 70 CFU could be indicative of infection by *Malassezia* spp. (Cafarchia et al. 2005b; Bajwa 2017).

All samples with a positive cytological examination had growth, except the sample 19 that showed a low cell count on the tape strip but had no growth. All the samples with a negative cytology showed no growth in any of the culture media used. Thus, cytological examination is a useful technique for diagnosing *Malassezia* pododermatitis (Cafarchia et al. 2005a; Duclos 2013). Modified Dixon’s agar has been suggested as the preferred media to culture *M. pachydermatis* due to the rapid growth of the colonies and its potential to support the lipid-dependent isolates that exist (Puig et al. 2017; Bond et al. 2020). However, in our study no significant differences were obtained in terms of CFU between SGA and mDA.

In our study, two cytological examinations were done using completely different techniques. In all cases, the tape strip method showed the highest abundance of cells, in agreement with other studies (Bond et al. 2020). A study on dogs with *Malassezia* pododermatitis showed that the number of yeasts recovered with swabs were significantly lower than those obtained by using other techniques as tape stripping, superficial scraping, and direct impressions (Miller et al. 2000).

Although a low number of samples has been analyzed, ITS copies of *M. pachydermatis* were increased on most of the samples of the pododermatitis group (73.3%) compared to that of healthy and post-treatment groups. Besides, the qPCR values dramatically decreased in samples from dogs after effective treatment. Due to the higher sensitivity of the qPCR method, DNA of *M. pachydermatis* was detected in all samples. As cytological examination is considered a useful technique for diagnosing *Malassezia* pododermatitis, the results of both cytological examinations were compared to those obtained by qPCR. A good correlation was observed between both diagnostic methods. These results showed that this qPCR method could be a fast useful technique to reliably detect *Malassezia pachydermatis* pododermatitis in dogs.

The fungal diversity studied using NGS was reduced in samples of the pododermatitis group compared with the healthy and post-treatment samples. These results are in accordance with other studies where a reduction of fungal species diversity and richness was observed in samples from dogs with otitis when compared to healthy dogs (Korbelik et al. 2018) and after treatment (Puigdemont et al. 2021). Also, atopic dogs usually present lower diversity in their microbiome when compared to healthy animals (Meason-Smith et al. 2015).

The results obtained in the metagenomic analysis were different between the pododermatitis samples and the healthy and post-treatment samples. *Basidiomycota* was the main phylum identified on pododermatitis samples while *Ascomycota* was the main one on healthy and post-treatment samples. Within the pododermatitis group, *Malassezia* was the majority genus identified in all three samples, and *M. pachydermatis* the main species. The genus *Malassezia* was also present in the healthy and post-treatment samples in a low abundance as it is a common member of the skin of dogs. Within the healthy and post-treatment samples, *Cladosporium* was the main genus followed by *Alternaria*. These genera are considered transient mycobiota as they are commonly isolated from environmental samples.

Our results agree with those obtained in studies of the ear and skin mycobiota of healthy dogs and dogs diagnosed with otitis or skin pathologies. In all these studies the main phylum identified in healthy dogs was *Ascomycota* and the main genus was part of the transient mycobiota. The second main genus identified in healthy dogs was *Malassezia*. In dogs diagnosed with otitis or dermatitis, *Basidiomycota* was the main phylum and *Malassezia* the main genus identified. *Malassezia pachydermatis* was the main species identified in dogs with otitis and atopic dermatitis (Korbelik et al. 2018; Bradley et al. 2020; Puigdemont et al. 2021). A study conducted by Tang et al. (2020) identified *M. pachydermatis* as the only yeast species present in the samples of both healthy dogs and dogs with otitis/dermatitis. These results differ from those obtained by Meason-Smith et al. (2020) where *M. restricta* was the main species detected in healthy and allergic canine skin. However, in this paper only molecular techniques were used, so this species was not isolated from those animals.

Focusing only on *Malassezia*, *M. pachydermatis* was the main species detected in all the samples, but *M. restricta, M. globosa, M. sympodialis* were also detected. *Malassezia restricta* and *M. globosa* have never been isolated from dogs (Bond et al. 2010). Although *M. sympodialis* has been previously cited from dogs with dermatitis and/or otitis, its identification was based solely on phenotypic characteristics (Raabe et al. 1998; Nardoni et al. 2004; Cafarchia et al. 2005a). Thus, the presence of small amounts of these three species in our samples could be a result of cross-contamination. These species are commonly found on the skin of healthy humans (Sugita et al. 2010) and thus, the contamination may have occurred during the sample extraction or contamination of the kit reagents, or they may be on the dog’s skin as a result from interaction with humans or with the indoor environment (Frau et al. 2019; Díaz et al. 2021).

Metagenomic studies on dog skin selected the ITS region as target gene. Although the ITS region is considered the universal barcode for fungi (Schoch et al. 2012), its variability among fungal species could lead to an incorrect estimation of fungal populations (Scorzetti et al. 2002; De Filippis et al. 2017). The LSU selected in this study as target gene has shown greater biodiversity in biological samples and works better for species discrimination in *Basidiomycota* and yeasts, especially within *Malassezia* genus (Hoggard et al. 2018; Mota-Gutiérrez et al. 2019). The use of this region allowed us the description of fungal diversity and the taxonomic classification of several *Malassezia* species. There was a percentage of unidentified *Malassezia* species within the pododermatitis (6.29%) and the healthy/post-treatment (0.05%) group. However, all the sequences belonged to the class *Malasseziomycetes* and could represent new taxa that have not been yet described.

The major limitation of this study was the sample size. Due to the high cost of the method, a total of five samples were selected for metagenomic analysis. Future studies with a larger sample size would be needed to clarify some of the results obtained in this study. Also, further studies including more breeds would be of interest to study the genetic predisposition of some breeds to *Malassezia* pododermatitis.

The NGS analysis allowed a better understanding of dynamics of *M. pachydemartis* pododermatitis. There is a difference in the results obtained in terms of fungal species identified and richness of fungal populations between healthy and post-treatment samples and pododermatitis samples. In the pododermatitis group *Malassezia* is predominant and in the healthy and post-treatment samples transient mycobiota predominates. Also, statistically significant differences were observed between dogs with pododermatitis before and after treatment and healthy dogs in values of cytological examination, CFU and qPCR. The results obtained showed a good correlation between cytologic and molecular methods and its value as a diagnostic tool.

## Supplementary Information

Below is the link to the electronic supplementary material.

Supplementary file1 (DOCX 13 kb)

## Data Availability

The raw sequencing data is available at the NCBI database (SRA accession number: PRJNA742914).

## References

[CR1] Bajwa J (2016). Canine pododermatitis. Can Vet J.

[CR2] Bajwa J (2017). Cutaneous cytology and the dermatology patient. Can Vet J.

[CR3] Bond R, Collin NS, Lloyd DH (1994). Use of contact plates for the quantitative culture of *Malassezia pachydermatis* from canine skin. J Small Anim Prac.

[CR4] Bond R, Guillot J, Cabañes FJ (2010). *Malassezia* yeasts in animal disease. *Malassezia* and The Skin.

[CR5] Bond R, Morris DO, Guillot J, Bensignor EJ, Robson D, Mason KV, Kano R, Hill PB (2020). Biology, diagnosis and treatment of *Malassezia* dermatitis in dogs and cats Clinical Consensus Guidelines of the World Association for Veterinary Dermatology. Vet Dermatol.

[CR6] Bradley CW, Lee FF, Rankin SC, Kalan LR, Horwinski J, Morris DO, Grice EA, Cain CL (2020). The otic microbiota and mycobiota in a referral population of dogs in eastern USA with otitis externa. Vet Dermatol.

[CR7] Cabañes FJ (2021). Diagnosis of *Malassezia* dermatitis and otitis in dogs and cats, is it just a matter of counting?. Rev Iberoam Micol.

[CR8] Cafarchia C, Gallo S, Capelli G, Otranto D (2005). Occurrence and population size of *Malassezia* spp. in the external ear canal of dogs and cats both healthy and with otitis. Mycopathologia.

[CR9] Cafarchia C, Gallo S, Romito D, Capelli G, Chermette R, Guillot J, Otranto D (2005). Frequency, body distribution, and population size of *Malassezia* species in healthy dogs and in dogs with localized cutaneous lesions. J Vet Diagn Invest.

[CR10] De Filippis F, Laiola M, Blaiotta G, Ercolini D (2017). Different amplicon targets for fungal sequencing-based studies of fungal diversity. Appl Environ Microbiol.

[CR11] Díaz L, Castellá G, Bragulat MR, Martorell J, Paytuví-Gallart A, Sanseverino W, Cabañes FJ (2021). External ear canal mycobiome of some rabbit breeds. Med Mycol.

[CR12] Duclos D (2013). Canine pododermatitis. Vet Clin North Am Small Anim Pract.

[CR13] Forbes JD, Knox NC, Ronholm J, Pagotto F, Reimer A (2017). Metagenomics: the next culture-independent game changer. Front Microbiol.

[CR14] Forde BM, O’Toole PW (2013). Next-generation sequencing technologies and their impact on microbial genomics. Brief Funct Genomics.

[CR15] Frau A, Kenny JG, Lenzi L, Campbell BJ, Ijaz UZ, Duckworth CA, Burkitt MD, Hall N, Anson J, Darby AC (2019). DNA extraction and amplicon production strategies deeply inf luence the outcome of gut mycobiome studies. Sci Rep.

[CR16] Giampaoli S, De Vittori E, Frajese GV, Paytuví A, Sanseverino W, Anselmo A, Barni F, Berti A (2020). A semi-automated protocol for NGS metabarcoding and fungal analysis in forensic. Forensic Sci Int.

[CR17] Guého E, Midgley G, Guillot J (1996). The genus *Malassezia* with description of four new species. Antonie Van Leeuw.

[CR18] Guého-Kellerman E, Boekhout T, Begerow D (2010). Biodiversity, phylogeny and ultrastructure. *Malassezia* and The Skin.

[CR19] Guillot J, Bond R (1999). *Malassezia pachydermatis*: a review. Med Mycol.

[CR20] Hoggard M, Vesty A, Wong G, Montgomery JM, Fourie C, Douglas RG, Biswas K, Taylor MW (2018). Characterizing the Human Mycobiota: A Comparison of Small Subunit rRNA, ITS1, ITS2, and Large Subunit rRNA Genomic Targets. Front Microbiol.

[CR21] Korbelik J, Singh A, Rosseau J, Weese S (2018) Analysis of the otic mycobiota in dogs with otitis externa compared to healthy individuals. Vet Dermatol 29:417e138. 10.1111/vde.12665. 10.1111/vde.12665 30088292

[CR22] Li H, Durbin R (2010). Fast and accurate long-read alignment with Burrows-Wheeler transform. Bioinformatics.

[CR23] Love MI, Huber W, Anders S (2014). Moderated estimation of fold change and dispersion for RNA-seq data with DESeq2. Genome Biol.

[CR24] McMurdie PJ, Holmes S (2013). phyloseq: An R Package for reproducible interactive analysis and graphics of microbiome census data. PLoS ONE.

[CR25] Meason-Smith C, Olivry T, Lawhon SD, Hoffmann AR (2020). *Malassezia* species dysbiosis in natural and allergen-induced atopic dermatitis in dogs. Med Mycol.

[CR26] Meason-Smith C, Diesel A, Patterson AP, Older CE, Mansell JM, Suchodolski JS, Rodrigues Hoffmann A (2015) What is living on your dog’s skin? Characterization of the canine cutaneous mycobiota and fungal dysbiosis in canine allergic dermatitis. FEMS Microbiol Ecol 91:fiv139. 10.1093/femsec/fiv139 10.1093/femsec/fiv139 PMC4657189 26542075

[CR27] Miller WH, Griffin CE, Campbell KL (2000). Fungal skin diseases. Muller & Kirk's Small Animal Dermatology.

[CR28] Mota-Gutiérrez J, Ferrocino I, Rantsiou K, Cocolin L (2019). Metataxomic comparison between internal transcribed spacer and 26S ribosomal large subunit (LSU) rDNA gene. Int J Food Microbiol.

[CR29] Nardoni S, Mancianti F, Corazza M, Rum A (2004). Occurrence of *Malassezia* species in healthy and dermatologically diseased dogs. Mycopathologia.

[CR30] Puig L, Bragulat MR, Castellá G, Cabañes FJ (2017). Characterization of the species *Malassezia pachydermatis* and re-evaluation of its lipid dependence using a synthetic agar medium. PLosONE.

[CR31] Puig L, Castellá G, Cabañes FJ (2019). Quantification of *Malassezia pachydermatis* by real-time PCR in swabs from the external ear canal of dogs. J Vet Diagn Invest.

[CR32] Puigdemont A, D’Andreano S, Ramió-Lluch L, Cuscó A, Francino O, Brazis P (2021). Effect of an anti-inflammatory pomegranate otic treatment on the clinical evolution and microbiota profile of dogs with otitis externa. Vet Dermatol.

[CR33] Raabe P, Mayser P, Weiss R (1998). Demonstration of *Malassezia furfur* and *M. sympodialis* together with *M. pachydermatis* in veterinary specimens. Mycoses.

[CR34] Reynolds DR, Taylor JW (1993) The Fungal Holomorph: Mitotic, Meiotic and Pleomorphic Speciation in Fungal Systematics, 1st edn, CAB international, Surrey, UK

[CR35] Schoch CL, Seifert KA, Huhndorf S, Robert V, Spouge JL, Levesque CA, Chen W (2012). Nuclear ribosomal internal transcribed spacer (ITS) region as a universal DNA barcode marker for Fungi. Proc Natl Acad Sci U S A.

[CR36] Scorzetti G, Fell JW, Fonseca A, Statzell-Tallman A (2002). Systematics of basiodiomycetous yeasts: a comparison of large subunit D1/D2 and internal transcribed spacer rDNA regions. FEMS Yeast Res.

[CR37] Sugita T, Tajima M, Tsubuku H, Tsuboi R, Nishikawa A (2006). Quantitative analysis of cutaneous *Malassezia* in atopic dermatitis patients using real-time PCR. Microbiol Immunol.

[CR38] Sugita T, Boekhout T, Velegraki A, Guillot J, Hađina S, Cabañes FJ (2010). Epidemiology of *Malassezia*-related skin diseases. *Malassezia* and The Skin.

[CR39] Tang S, Prem A, Tjokrosurjo J, Sary M, Van Bel MA, Rodrigues-Hoffmann A, Kavanagh M, Wu G, Van Eden ME, Krumbeck JA (2020). The canine skin and ear microbiome: A comprehensive survey of pathogens implicated in canine skin and ear infections using a novel next-generation-sequencing-based assay. Vet Microbiol.

[CR40] Vuran E, Karaarslan A, Karasartova D, Turegun B, Sahin F (2014). Identification of *Malassezia* species from pityriasis versicolor lesions with a new multiplex PCR method. Mycopathologia.

