# Supplementary material for: Study of the variation of the Malassezia load in the interdigital fold of dogs with pododermatitis
Source: Vet Res Commun. 2022 Jun 15;47(2):385–96. doi: 10.1007/s11259-022-09951-2 (PMC10209231; doi:10.1007/s11259-022-09951-2)
Supplement: Supplementary file 1 — Supplementary file1 (DOCX 13 kb) [file 11259_2022_9951_MOESM1_ESM.docx]

Supplementary Table 1. Mean Cq values of samples.

| Sample | Dog | Mean Cq |
| --- | --- | --- |
| Samples from dogs with pododermatitis | | |
| 1 | Dog 1 | 26.30 |
| 2 | Dog 2 | 30.50 |
| 3 | Dog 3 | 27.23 |
| 4 | Dog 4 | 27.99 |
| 5 | Dog 5 | 23.00 |
| 6 | Dog 5 | 24.40 |
| 7 | Dog 6 | 26.71 |
| 8 | Dog 7 | 22.48 |
| 9 | Dog 8 | 30.64 |
| 10 | Dog 8 | 30.23 |
| 11 | Dog 9 | 23.43 |
| 12 | Dog 9 | 26.54 |
| 13 | Dog 10 | 22.57 |
| 17 | Dog 13 | 29.80 |
| 18 | Dog 13 | 28.17 |
| Samples from healthy dogs | | |
| 14 | Dog 11 | 28.61 |
| 15 | Dog 11 | 34.71 |
| 16 | Dog 12 | 30.70 |
| Samples after antifungal treatment | | |
| 19 | Dog 3 | 27.12 |
| 20 | Dog 7 | 32.49 |
| 21 | Dog 8 | 31.63 |
| 22 | Dog 8 | 33.78 |
